# Mendelian randomization analyses reveal no genetic causal effects of major adipokines on systemic lupus erythematosus

**DOI:** 10.1371/journal.pone.0301699

**Published:** 2024-05-28

**Authors:** Peng Duan, Suyan Tian

**Affiliations:** 1 Intensive Care Unit (ICU), The First Hospital of Jilin University, Changchun, Jilin, P.R. China; 2 Division of Clinical Research, The First Hospital of Jilin University, Changchun, Jilin, P.R. China; Charotar Institute of Applied Sciences: P D Patel Institute of Applied Sciences, INDIA

## Abstract

Epidemiological studies have shown that the levels of serum adipokine such as leptin and resistin are associated with the risk of developing systemic lupus erythematosus (SLE). Nevertheless, whether either leptin or resistin has causal impacts on the risk of SLE is still unknown. In this study, two-sample univariable MR analyses and multivariable MR analysis were performed to explore the causal relationships between adipokines and SLE. Additionally, the potential causal effects of SLE on major adipokines were evaluated using reverse MR analyses. The results of inverse-variance weighted (IVW), weighted median, weighted mode and MR‒Egger methods concordantly supported that major adipokines have no causal effects on the risk of SLE. In the multivariable MR IVW analysis with leptin and resistin as covariates, neither leptin (odds ratio (OR) = 3.093, *P* = 0.067) nor resistin (OR = 0.477, *P* = 0.311) was identified as an independent risk factor for SLE, which is in line with the univariable MR results. In conclusion, our analyses revealed no evidence to support that these three major adipokines are risk factors for SLE.

## Introduction

Systemic lupus erythematosus (SLE), a chronic autoimmune disease, is characterized by the activation of inflammatory immune cells and the production of proinflammatory autoantibodies that would influence multiple organs. Therefore, SLE is considered as a systemic syndrome rather than a single disease. The prevalence of SLE is estimated to be 30 to 150 per 100,000 people, and its incidence ranges from 2.2 to 23.1 per 100,000 people annually [1], imposing a heavy health burden on society and families. Therefore, the underlying mechanisms that lead to the onset and progression of the disease must be understood to determine effective treatment regimens for SLE.

Several adipokines synthesized in the adipose tissue, particularly adiponectin, leptin, and resistin, have been explored in patients with SLE, and the results suggest that they may be involved in the pathogenesis of SLE. For instance, leptin, a small polypeptide hormone secreted by the adipocytes, binds to a receptor located in the hypothalamus and can control body weight and gonadal function [2]. Leptin levels are highly correlated with adiposity, a commonly known risk factor for a variety of complex diseases such as diabetes, certain cancer types, and cardiovascular disease [3]. To date, observational studies and meta-analyses [4–7] have shown that compared to healthy controls, serum/plasma leptin levels are elevated in patients with SLE and tend to increase with disease severity. Furthermore, a review article [8] discussed the pathogenic mechanism of leptin in SLE to provide insights into the development of leptin-based approaches for the treatment of SLE. However, not all studies have reached concordant conclusions about the association between leptin and SLE; for example, a case-control study involving 60 newly diagnosed treatment naïve SLE patients and 40 age and sex-matched normal controls [9] found that leptin levels were significantly reduced in SLE patients.

Like leptin, resistin has been shown to play important roles in regulating weight and glucose metabolism. However, no concordant conclusions have been reached about the association between resistin and SLE yet. For instance, the results of a meta-analysis comprising of six independent studies, including 559 patients and 430 healthy controls, suggested that no significant difference in serum resistin levels existed between SLE patients and healthy controls [10]. A cross-sectional observational study concluded that SLE was significantly associated with higher resistin levels based on the results of a multivariable regression model [11].

For adiponectin, two contradicting examples were an observational study by [6] in which no significant difference between SLE patients and controls was found and by [12] in which high serum adiponectin was demonstrated to be related to accelerated carotid atherosclerosis in young SLE women. Notably, unmeasured confounding factors and reverse causation affect observational studies, making a causal inference of these three adipokines on SLE difficult or even impossible.

Mendelian randomization (MR) is a commonly used epidemiologic method in which either the summary statistics or individual data from GWAS studies can be employed. Genetic variants are utilized as instrumental variables (IVs) to detect whether an exposure has a causal impact on an outcome [13]. Additionally, to provide unbiased causal effect estimation, MR analysis helps improve the success rate of drug development [14]. Given that MR analysis possesses these outstanding merits, we conducted a two-sample MR study to examine whether three major adipokines have a causal impact on the risk of SLE.

## Material and methods

Two-sample MR analysis was conducted to explore whether adipokines have a causal impact on the development and progression of SLE using the R TwoSampleMR package [15]. In these analyses, each adipokine is regarded as the exposure, and SLE is the outcome. Independent single-nucleotide polymorphisms (SNPs) (with linkage disequilibrium *R*^*2*^ <0.001), associated with the exposure at the genome-wide significance (*P* < 5×10^−8^, which is the default cutoff value) were selected from the MR-Base GWAS catalog. Specifically, a GWAS dataset comprising 14 GWAS studies including 30,931 individuals [16] was used for resistin, a GWAS study including 57,232 individuals of European ancestry [17] was used for leptin, and a GWAS, i.e., the ADIPOGen consortium [18] including 39,883 individuals, was used for adiponectin.

For the outcome, we used the summary GWAS data from the FinnGen consortium. The FinnGen consortium aims to collect and analyse genome and health data from 500,000 Finnish Biobank participants, and the summary data used in this study were released on May 11,2021 (https://www.finngen.fi/en), which included 442 SLE patients and 218,254 controls of European ancestry.

In Mendelian randomization, SNPs must satisfy three assumptions to be considered as valid IVs. First, it is strongly correlated with exposure. Second, it must not be associated with any confounding factors. Finally, it affects the outcome only via the exposure, rather than via other pathways or through a direct impact on the outcome. A genetic variant violates either or both of the last two assumptions, it is referred to possess horizontal pleiotropy. In the literature, numerous MR methods have been proposed. Among them, the inverse-variance weighted (IVW) method is often considered as a primary method, in which the above three assumptions must be rigorously satisfied. In IVW, the effect of each SNP on the outcome was initially weighed by its effect on the exposure using the Wald ratio method, resulting in individual MR estimates. Then, these individual estimates were integrated into an overall summary value using a meta-analysis.

On the other hand, not all SNPs must be valid in other complementary MR methods, such as MR‒Egger [19], weighted mode [20], and weighted median [21] methods. Specifically, the weighted median method involves the assumption that at least 50% of the IVs are valid. As its name implies, this method uses a median weighted by the precisions of individual MR estimates as the overall effect. Similar to IVW, MR‒Egger regression also involves a weighted linear regression of SNP‒outcome associations on SNP–exposure associations, with an intercept term that can be utilized to evaluate the existence of directional pleiotropy. Thus, the essential difference between these two methods is whether the regression model includes an intercept. Finally, the weighted mode method utilizes the estimate based on the largest valid SNP cluster. Therefore, all SNPs in the remaining clusters can be invalid. In this study, IVW was also chosen as the primary method for estimating the genetic effect size of adipokine levels on SLE susceptibility. Moreover, analysis results using the MR‒Egger regression [19], the weighted median estimator [20], and the weighted mode method [21], which are more robust to horizontal pleiotropy, were also presented. If all four MR methods give the concordant results, the results are considered to be robust.

In addition, MR‒Egger intercept, the funnel plots, Cochrane’s Q statistics and leave-one-out sensitivity analyses were used to assess the existence of pleiotropy and heterogeneity. Finally, a multivariable MR (MVMR) analysis with adipokines as covariates was conducted. MVMR is an extension of standard MR. It is usually difficult to identify a unique and exclusive genetic variant associated with a particular exposure, although it is possible to identify a genetic variant associated with a set of exposure variables. In MVMR, those exposure variables are all considered as covariates to estimate their direct effects on the outcome. The R Mendelian Randomization package was utilized to perform MVMR analysis.

A power calculation was conducted using an online calculator, namely, mRnd (https://cnsgenomics.com/shiny/mRnd) [22] to assess whether the statistical power of the current univariable MR analysis was adequate. The current study was prepared with reference to the STrengthening the Reporting of Observational studies in Epidemiology-MR (STROBE-MR) checklist [23].

## Results

### Power calculation

First, we conducted a post-hoc power calculation to evaluate if the current MR study had adequate statistical power. At the significance level of 0.05, a sample size of 218,696 (the sample size of the FinnGen study specifically for SLE) provides a power of 0.44 to detect a causal risk of 1.5 (OR = 1.5) to develop SLE when the genetically predicted leptin/resistin/adiponectin level increases by one unit. It was assumed that these genetic variants explained approximately 3% of the variance in the leptin/resitin/adiponectin level. This calculation indicates that the current MR study may be insufficiently statistically powered for a subtle causal risk.

### MR results for leptin

Various methods were used to conduct a thorough examination of pleiotropic effects. According to MR‒Egger regression, no pleiotropic effects (the intercept: 0.18, *P* = 0.377) were found among the 6 leptin-associated SNPs. There was no heterogeneity among these SNPs (Cochran’s Q value = 4.37, *P* = 0.358 for MR Egger, and Cochran’s Q value = 5.45, *P* = 0.364 for IVW). Moreover, the funnel plot revealed no discernible heterogeneity or extreme outliers among the six SNPs. Hence, based on the results of these statistical tests, the SNPs used in the current MR analysis were valid IVs.

**Fig 1:**
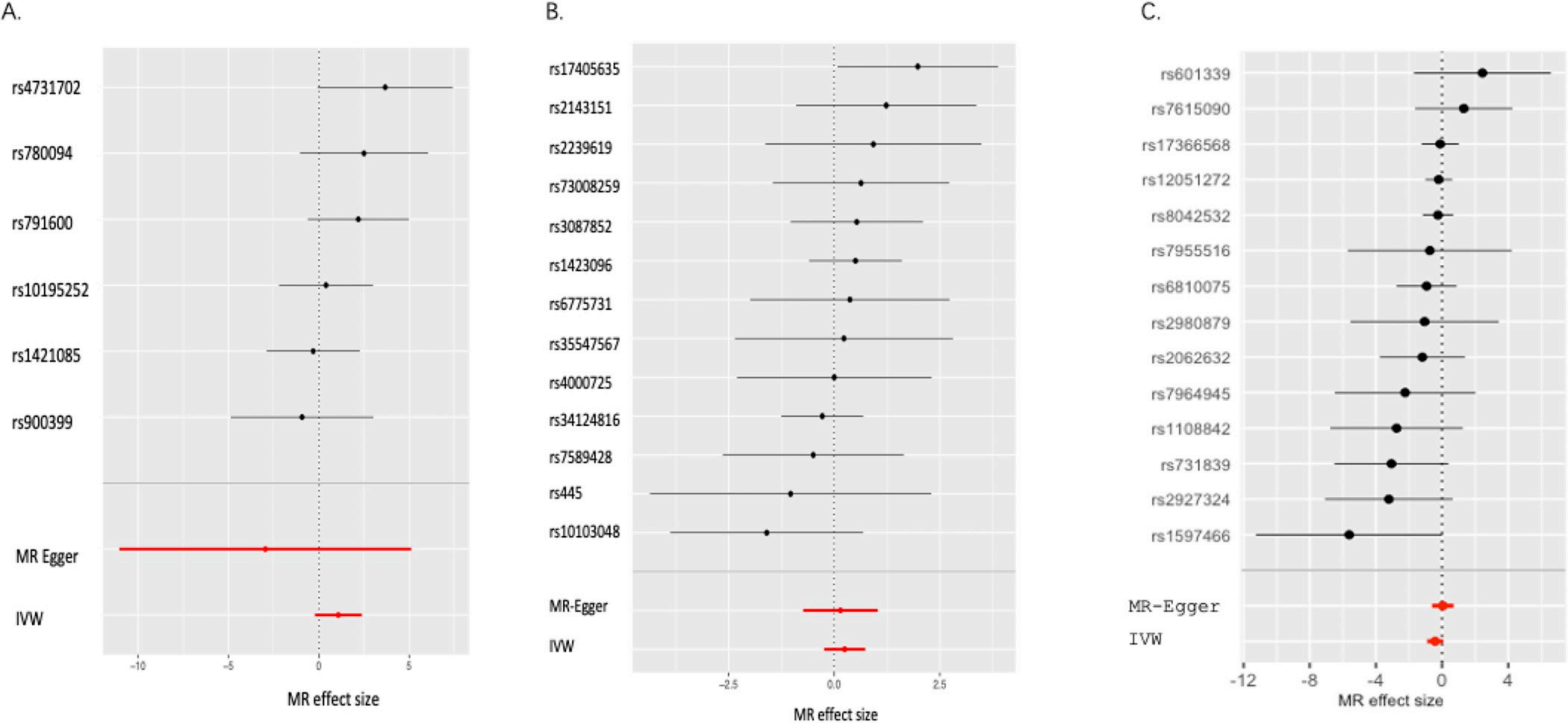
Forest plots of univariable Mendelian randomization analyses to examine the causal effects of circulating adipokine levels on the risk of systemic lupus erythematosus. A) For leptin. B) For resistin. C) For adiponectin.

Based on the six leptin-associated SNPs, no causal relationship between leptin and SLE was discovered in the main analysis using the IVW method. The odds ratio (OR) of developing SLE per one unit increment of genetically predicted leptin level was estimated to be 2.941 (*P* = 0.105) (**Fig 1A**). The weighted median, weighted mode and MR‒Egger methods’ results confirmed the robustness of leptin’s null causal effect on the risk of SLE, as they were generally similar to the main analysis using IVW method. The ORs were estimated to be 0.052 (*P* = 0.512), 2.041 (*P* = 0.396), and 1.167 (*P* = 0.902) by the MR‒Egger, weighted median and weighted mode methods, respectively. Nonetheless, the IVW, weighted median and weighted mode methods estimated the association coefficients as positive values (a potential hazard factor despite being insignificant), whereas the MR‒Egger method produced a negative coefficient (a protective factor despite of being insignificant). Furthermore, the extreme estimated value and inflated confidence intervals derived using MR‒Egger suggested that the results should be interpreted with extreme caution. Finally, funnel plot and leave-one-out sensitivity analysis identified no outliers that displayed heterogeneity and thus had a significant impact on effect estimation.

### MR results for resistin

Likewise, neither pleiotropic effects nor outliers were found by any of the statistical tests, including MR‒Egger intercept, Q statistics, LOO analysis and a funnel plot for resistin. Based on the 13 resistin-associated SNPs, no causal relationship between resistin and SLE was revealed in the main analysis using the IVW method. The OR of developing SLE per one unit increment of genetically predicted resistin level was estimated to be 1.280 (*P* = 0.322) (**Fig 1B**). The weighted median, weighted mode and MR‒Egger methods’ results confirmed the robustness of leptin’s null causal effect on the risk of SLE, as they were generally similar to the main analysis using IVW method. The ORs were estimated to be 1.160 (*P* = 0.749), 1.488 (*P* = 0.241), and 1.375 (*P* = 0.477) by the MR‒Egger, weighted median and weighted mode methods, respectively. In contrast to leptin, all estimates given by the four methods are positive despite being insignificant.

### MR results for adiponectin

Based on the 14 adiponectin-associated SNPs, no causal relationship between adiponectin and SLE was revealed by the IVW method. The OR of developing SLE per one unit increment of genetically predicted adiponectin level was estimated to b3 0.660 (*P* = 0.097) (**Fig 1C**). Again, neither pleiotropic effects nor outliers were found by the MR‒Egger intercept term, Q statistics, LOO analysis, and funnel plot for adiponectin.

The weighted median, weighted mode and MR‒Egger methods’ results confirmed the robustness of the lack of a causal effect of adiponectin on the risk of SLE. Specifically, the ORs were estimated to be 1.047 (*P* = 0.892), 0.805 (*P* = 0.467), and 0.765 (*P* = 0.304) by the MR‒Egger, weighted median and weighted mode methods, respectively. Consistent results, namely, null causal genetic effect of adiponectin on SLE, were obtained by another MR study [24], despite different GWAS studies being used. With such strong evidence to support this null effect, we decided not to include adiponectin in the multivariable MR analysis.

### Reverse MR results

To assess whether reverse causality existed between these three major adipokines and SLE, we used the same GWAS studies to perform reverse MR analyses. For leptin, the IVW method indicated a null reverse causal relationship based on two SLE-associated SNPs. The other three MR methods were not suitable since the number of SNPs was less than three. In contrast, while all four MR methods supported a null reverse causal relationship between resistin and SLE, the results of adiponectin were conflicting, namely, both IVW (OR = 0.971, *P* = 0.021) and weighted mode (OR = 0.971, *P* = 0.026) suggested a negative causal association; both MR‒Egger (OR = 1.017, *P* = 0.830) and weighted median (OR = 0.970, *P* = 0.142) supported a null reverse causal relationship. Nevertheless, the results of all four MR methods became consistent with each other when the correction for multiple comparisons was corrected.

### Multivariable MR results

Finally, using the same GWAS studies, a multivariable MR analysis including both lepin and resistin as covariates was conducted in which only the IVW method was considered. Neither leptin (OR = 3.093, *P* = 0.067) nor resistin (OR = 0.477, *P* = 0.311) was again identified as an independent risk factor for SLE. Interestingly, the association direction between resistin and SLE became negative (despite remaining non-significant) instead after controlling for circulating leptin levels.

## Discussion and conclusion

Adipocytes are not only a repository of energy in the form of fat but also an important source of adipokines [25]. Adipokines are neuromodulators, growth factors, proteins of the complement system, acute phase proteins, stress hormones, and proteins involved in glucose homeostasis. Changes in adipokine levels may impair the balance between proinflammatory and anti-inflammatory cytokines [26], potentially leading to diverse autoimmune diseases, such as SLE and psoriasis. Numerous studies, including observational studies, clinical trials, and animal experiments have been performed to examine the association between adipokine levels and SLE risk. As mentioned, above however, the existing studies [4–12] on the associations between the three major adipokines and SLE have shown inconsistent results; some support null associations while others support significantly correlated relationships. Since the observational studies are usually subject to confounding effects (either unobserved or unadjusted) and reverse causality, especially for retrospective studies, the positive findings of these previous studies should be interpreted with caution. It is worth noting that these existing studies commonly have several disadvantages, such as a small to moderate sample size, the use of conventional statistical methods, and the collection of samples from a single center; thus, these studies are incapable of providing a good representation on the whole population. In contrast, a MR study suffers marginally from both confounding effects and reverse causality. Moreover, it provides a causal inference, capable of distinguishing between a cause and a result, which is promising in the clinic.

However, we acknowledge that the current study has two major limitations. First, the sample size of this MR analysis was moderate and therefore might be incapable of detecting a subtle effect. Therefore, the null causal effects obtained in the current MR study may be a result of the insufficient statistical power. Further investigation is needed. The second limitation is that the study populations of both GWAS summary datasets were different. The news and blogs on the internet have reported that the Finnish population is genetically different from European ancestry with respect to linkage disequilibrium (LD) structure and allele frequency, possibly producing a biased estimation of causal effects (including the scenario in which significant effects were biased toward null effects). Additionally, some remarked that the Finnish population more closely resembles to the Asian population. Correspondingly, we reran a two-sample MR analysis, replacing the FinnGen study with a GWAS study of 512 SLE patient and 994 matched controls from the Chinese population [27]. The corresponding results indicated the null causal relationship between circulating leptin/resistin/adiponectin levels and the risk of developing SLE remained unchanged. It is worth pointing out that the GWAS study based on the Chinese population had a very small sample size. Since the FinnGen study, whose sample size is relatively large compared to this study, has been shown to possess inadequate statistical power, the sample size is a major concern for this analysis based on the Chinese population and the null results should be interpreted with caution.

Overall, the current study did not provide explicit/direct evidence of either leptin or resistin or adiponectin being a risk factor for SLE. To the best of our knowledge, the present study is the first to utilize both two-sample univariable MR and multivariable MR methods to evaluate the causal relationship between major adipokines and SLE. The MR study design can infer causality, being listed before an observational study for the evidence priority [28]. Therefore, we concluded that the associations between adipokines and SLE observed in those epidemiological studies are highly likely to result from remaining or unobserved confounding effects. Furthermore, since the current study has limitations in terms of sample size and population ethnicity, large-sized and cross-race genomics and prospective observational studies are highly desirable.
